# Effects of changes in regular physical activity status on hip fracture: A nationwide population-based cohort study in Korea

**DOI:** 10.1371/journal.pone.0249819

**Published:** 2021-04-08

**Authors:** Sangsoo Han, Hae-Dong Jang, Sangun Nah, Kyungdo Han, Hyunwoong Lim, Won Seok Kim, Jae-Young Hong

**Affiliations:** 1 Department of Emergency Medicine, Soonchunhyang University Bucheon Hospital, Bucheon, Republic of Korea; 2 Department of Orthopaedic Surgery, Soonchunhyang University Bucheon Hospital, Bucheon, Republic of Korea; 3 Department of Biostatistics, College of Medicine, Catholic University, Seoul, Republic of Korea; 4 Department of Industrial Management Engineering, Korea University, Seoul, Republic of Korea; 5 Department of Orthopedics, Korea University Hospital, Ansan, Gyeonggi-do, Republic of Korea; Medical College of Wisconsin, UNITED STATES

## Abstract

**Objective:**

Hip fracture incidence is increasing with rapid aging of the population and regular physical activity (RPA) is an important modifiable protective factor for fracture. However, the association between the risk of hip fractures and changes in RPA status in the general population remains unknown. Thus, we explore the association between the risk of hip fracture and changes in RPA status.

**Methods:**

We studied 4,984,144 individuals without fractures within a year whose data were registered in the Korean National Health Insurance Service database. Baseline physical activity level was assessed using a standardized self-reported questionnaire during two consecutive national health screening surveys performed in Korea from 2009 to 2012. The risk of hip fracture between 2013 and 2016 according to change in RPA was prospectively analyzed. Participants were divided into those who were always inactive, became inactive, became active, and were always active.

**Results:**

Compared to participants who were always inactive, those who became inactive exhibited a 0.12/1,000 person-years (PY) reduction in hip fracture incidence rate (IR) [aHR: 0.865; 95% confidence interval (CI): 0.824–0.908]. Participants who became active, and those who were always active, exhibited a 0.24/1,000 PY reduction in IR (aHR: 0.827; 95% CI: 0.787–0.870) and a 0.39/1,000 PY reduction in IR (aHR: 0.691; 95% CI: 0.646–0.740), respectively.

**Conclusion:**

Changes in RPA status were associated with the risk of hip fracture; consistent RPA was related to the maximum benefit for risk reduction in the general population.

## Introduction

A hip fracture is a serious fracture for which the risk increases with age. A hip fracture is more complex than fracture of an extremity (e.g., the wrist) because a patient with hip fracture often cannot live independently; a hip fracture increases morbidity and mortality (by approximately 25%) within 1 year after fracture [1–3]. Thus, hip fracture is both a socioeconomic and personal problem. Patients with hip fracture may be unable to engage in economic activity prior to recovery. Notably, the fracture incidence is increasing with rapid aging of the population [4].

It is important to prevent hip fractures by enhancing bone strength or reducing the risk of falls. Risk factors for hip fracture include a poor diet low in calcium, cigarette smoking, physical inactivity, poor housing, and heavy alcohol use [5]. Of these risk factors, physical inactivity is an important modifiable factor. However, the prevalence of physical inactivity is high; only 16% of individuals aged ≥ 65 years in the United States are physically active, as determined by questionnaires based on recognized aerobic and muscle-strengthening guidelines [6]. Fewer than one-third of European adults engage in at least 150 min of physical activity weekly [6].

Physical activity is any bodily movement created by skeletal muscle(s) associated with energy expenditure [7]. To prevent fractures, moderate-to-vigorous physical activity (MVPA) is required; light physical activity (during daily life) is inadequate. Trimpou et al. found that high-intensity physical activity reduced the risk of hip fracture [8]. Englund et al. found that MVPA reduced hip fractures, whereas low-intensity physical activity (commuting, occupational activity, training, or cycling) did not [9]. Thus, we explored the relationship between the risk of hip fractures and changes in regular physical activity (RPA) (moderate physical activity for at least 30 min on at least 5 days per week or vigorous physical activity for at least 20 min on at least 3 days per week) as recommended by public health guidelines [10]. To the best of our knowledge, no study has yet reported any relationship between changes in RPA status and the risk of hip fracture.

In the present study, we used nationwide population-based data from Korea recorded from 2009 to 2012 to explore the association between the risk of hip fracture and changes in RPA status. We sought to characterize the relationship between changes in RPA status and subsequent hip fractures, and to determine whether this association differed according to sex, age, chronic conditions (e.g., diabetes), and prior fracture.

## Materials and methods

### Study population and data source

This nationwide, observational cohort study was based on claims data of the National Health Insurance Service (NHIS). All insured Koreans aged ≥ 40 years, and all workers aged ≥ 20 years, must undergo regular NHIS checkups every 1–2 years. The NHIS is a quasi-governmental agency established by the Ministry of Health and Welfare to provide healthcare for > 97% of Koreans. The NHIS database is an invaluable resource for population-based cohort studies; it contains demographic, health screening, diagnostic, and drug prescription data, which are collected on a regular basis and carefully quality-controlled.

**Fig 1:**
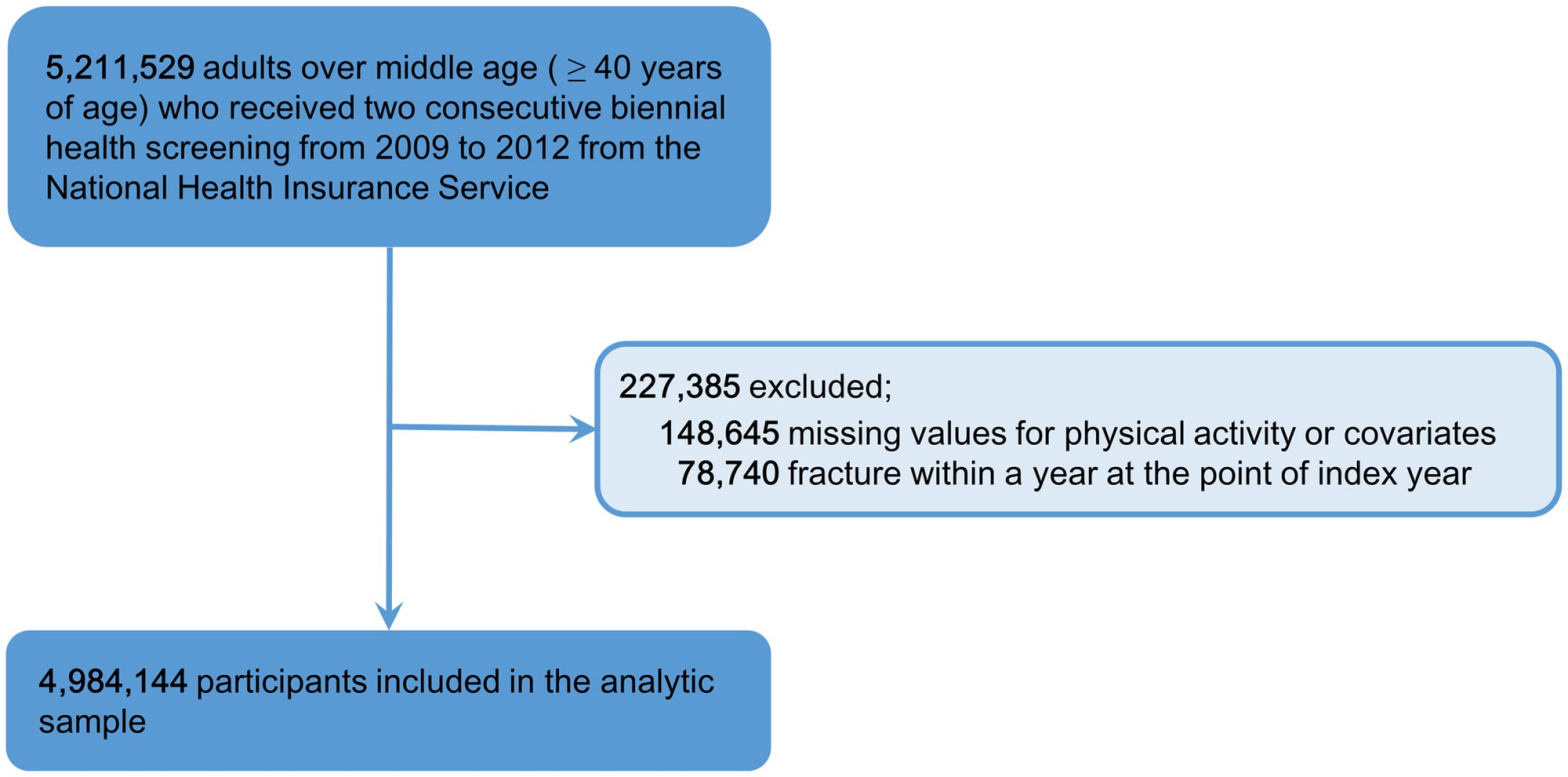
Flow diagram of study population selection.

We enrolled 5,211,529 individuals aged ≥ 40 years who underwent two consecutive biennial NIHS health screens during two periods: either 2009 and 2010, or 2011 and 2012. We excluded individuals for whom physical activity data were lacking, or who experienced fractures within the prior year (Fig 1).

The study protocol was approved by the NHIS Institutional Review Board. Informed consent from participants was not required, because the NHIS data are anonymized. The study protocol was also approved by the Institutional Review Board of Korea University Hospital (Ansan, South Korea; approval no. 2020AS0030).

### Changes in regular physical activity

During both screening periods, all participants completed questionnaires exploring physical activity and other lifestyle behaviors. We used the International Physical Activity Questionnaire (IPAQ) short version [11] to explore physical activity intensity, based on the NHIS survey responses. The IPAQ features seven questions exploring the frequency and duration of physical activity (thus activity intensity) over the previous seven days. We recorded the numbers of moderate activities (≥30 min per day; e.g., walking, dancing, gardening) and vigorous physical activities (≥20 min per day; e.g., running, rapid cycling, aerobics) per week during both 2009–10 and 2011–12; we sought to determine changes in RPA levels. We defined RPA as vigorous physical activity on ≥ 3 days per week or moderate physical activity on ≥ 5 days per week. We defined RPA changes from the first (2009–10) to the second (2011–12) examination as follows: 1) always inactive (non-RPA to non-RPA); 2) becoming inactive (RPA to non-RPA); 3) becoming active (non-RPA to RPA); and 4) always active (RPA to RPA).

### Hip fracture follow-up

We used the medical claims records of the NHIS to identify hip fracture events during the follow-up period from January 1, 2013 to December 31, 2016; we examined hospitalization records and the codes of the International Classification of Diseases Tenth Revision (ICD-10). We defined a hip fracture as hospitalization diagnostic codes of S72.0, S72.1, and S72.2. Participants who died during follow-up were censored at the time of death.

### Variables used for adjustment and subgroup analyses

We collected socioeconomic data (age, sex, and household income) and information regarding comorbidities by examining NHIS insurance eligibility and medical insurance claim databases. The NHIS National Health Screening database contains data regarding lifestyle behaviors (smoking and alcohol consumption); laboratory test results (total cholesterol and fasting serum glucose levels); and clinical data (body mass index [BMI], blood pressure, and estimated glomerular filtration rate). The study population was subdivided into two groups according to age (40–64 years and ≥ 65 years) and BMI (< 25 and ≥ 25 kg/m^2^). Low income was defined as an income below the 20^th^ percentile. Prior fracture was defined as a fracture that occurred within 2–3 years prior to the index year. Comorbidity definitions based on ICD codes have been validated in previous studies [12, 13]. Diabetes was identified by prescription of anti-diabetic drugs with ICD-10 codes E11–E14 or a fasting blood glucose level > 126 mg/dL; hypertension was identified by systolic/diastolic blood pressures ≥ 140/90 mmHg or at least one annual claim for an antihypertensive agent with ICD-10 codes I10–I13 or I15; dyslipidemia was identified by a total cholesterol level ≥ 240 mg/dL or at least one annual claim for an antihyperlipidemic agent with ICD-10 code E78; and chronic kidney disease (CKD) was identified by an estimated glomerular filtration rate < 60 mL/min/1.73 m^2^.

### Statistical analyses

We used the chi-squared test to compare categorical variables and Student’s t-test to compare continuous variables. The incidence rate (IR) was the outcome rate per 1,000 person-years (PY), divided by the total number of hip fractures. We calculated the hazard ratios (HRs) and 95% confidence intervals (CIs) for hip fractures according to RPA status at a single timepoint by using Cox regression analysis. We employed a Cox proportional hazards regression model to evaluate the association between hip fracture risk and change in RPA status from the first to the second biennial national health screen (2009–10 to 2011–12). We constructed four models to explore covariates potentially associated with hip fractures. Model 1 was unadjusted. Model 2 was adjusted for age and sex. Model 3 was additionally adjusted for smoking status, alcohol consumption, and household income. Model 4 was additionally adjusted for BMI, diabetes status, and fractures within the prior 3 years. Model 5 was fully adjusted, containing additional adjustments for region.

To explore the impacts of clinical conditions on the association between change in RPA status and risk of hip fracture, the HRs for hip fractures in various subgroups were derived via Cox regression analysis, as were P-values for interaction. We performed stratified subgroup analysis by sex; age (< 65 and ≥ 65 years); BMI (< 25 and ≥ 25 kg/m^2^); household income (< 20^th^ and ≥ 20^th^ percentile); smoking status (current and never); alcohol consumption (none and current); comorbidities (diabetes, hypertension, dyslipidemia, and CKD); and prior fracture status (within 3 years or not). All statistical analyses were performed using SAS software (ver. 9.3; SAS Institute, Cary, NC, USA). A two-sided P-value <0.05 was considered to indicate statistical significance.

## Results

### Baseline characteristics

The average age of the 4,984,144 participants was 54.9±10.1 years; 2,659,029 (53.3%) were men. Of all participants, 3,303,504 (66.3%) were always inactive, 583,561 (11.7%) became inactive, 650,888 (13.1%) became active, and 446,191 (9.0%) were always active. Participant demographic and clinical characteristics are listed in Table 1. The proportions of men, participants aged ≥ 65 years, participants with high BMI, current smokers and drinkers; low-income participants; those with comorbidities such as diabetes, hypertension, dyslipidemia, and CKD; and participants with prior fracture history, differed significantly among the groups, as did mean age and BMI (all P<0.0001). All parameters differed significantly according to change in RPA status; the study population was very large. During follow-up, the prevalences of newly diagnosed hip fracture were 0.4% (n = 12,237) in the always inactive group, 0.3% (n = 1,873) in the group that became inactive, 0.3% (n = 1,745) in the group that became active, and 0.2% (n = 928) in the always active group. The incidence was thus highest in the always inactive group. The results of post-hoc analysis are presented in S1 Table.

**Table 1:** Baseline characteristics stratified by change in regular physical activity.

	Always inactive	Became inactive	Became active	Always active	P-value
	(n = 3,303,504)	(n = 583,561)	(n = 650,888)	(n = 446,191)	
Male sex, n (%)	1,687,642 (51.1)	322,733 (55.3)	360,244 (55.4)	288,410 (64.6)	<0.0001
Age, years	54.7 ± 10.5	56.0 ± 10.2	55.0 ± 9.91	55.1 ± 9.7	<0.0001
≥ 65 (%)	623,067 (18.9)	124,656 (21.4)	120,002 (18.4)	81,934 (18.4)	<0.0001
BMI, kg/m2	23.9 ± 3.1	24.2 ± 2.9	24.0 ± 2.9	24.2 ± 2.8	<0.0001
≥25 (%)	1,120,785 (33.9)	212,040 (36.3)	223,493 (34.3)	159,046 (35.7)	<0.0001
Current smoker (%)	701,111 (21.2)	105,362 (18.1)	116,059 (17.8)	76,412 (17.1)	<0.0001
Current drinker (%)	1,391,493 (42.1)	250,991 (43.0)	293,956 (45.2)	231,673 (51.9)	<0.0001
Low income (%)	679,813 (20.6)	121,334 (20.8)	137,779 (21.2)	82,003 (18.4)	<0.0001
Comorbidities (%)					
Hypertension	1,094,622 (33.1)	212,878 (36.5)	224,188 (34.4)	158,971 (35.6)	<0.0001
Diabetes	378,394 (11.5)	79,911 (13.7)	82,294 (12.6)	59,516 (13.3)	<0.0001
Dyslipidemia	820,600 (24.8)	156,172 (26.8)	166,169 (25.5)	114,208 (25.6)	<0.0001
CKD	203,313 (6.2)	38,170 (6.5)	40,453 (6.2)	28,153 (6.3)	<0.0001
Urban region (%)	1,429,941 (43.3)	266,785 (45.7)	305,875 (47.0)	223,557 (50.1)	<0.0001
Prior fracture (%)	99,987 (3.0)	17,532 (2.7)	17,376 (3.0)	10,148 (2.3)	<0.0001
Hip fracture (%)	12,237 (0.4)	1,873 (0.3)	1,745(0.3)	928 (0.2)	<0.0001

BMI, body mass index; CKD, chronic kidney disease.

### Association between RPA and hip fracture

Over the 20.1 million PY of follow-up, we recorded 16,783 hip fractures. After multivariable adjustment (Model 5, adjusted for age, sex, smoking status, alcohol consumption, household income, BMI, diabetes, prior fracture and region), the RPA group exhibited a significantly reduced risk of hip fracture, compared to the non-RPA group (0.29/1,000 PY reduction in IR; adjusted HR [aHR]: 0.795; 95% CI: 0.763–0.829). Thus, RPA appeared to prevent hip fracture (Table 2).

**Table 2:** Risk of hip fracture according to regular physical activity in the National Health Insurance Service cohort screened in 2011 and 2012.

Group	Fracture events (n)	Total FU duration (PY)	IR (per 1,000 PY)	Hazard ratio (95% CI)				
				Model 1	Model 2	Model 3	Model 4	Model 5
Non-RPA	14,110	16,498,793.38	0.86	1	1	1	1	1
RPA	2,673	4,694,649.23	0.57	0.667	0.762	0.783	0.792	0.795
				(0.640–0.695)	(0.731–0.795)	(0.751–0.816)	(0.760–0.826)	(0.763–0.829)

FU, follow-up; PY, person-year; IR, incidence rate; CI, confidence interval; PRA, regular physical activity.

Incidence rate = Fracture event/total follow-up duration.

Model 1: Non-adjusted.

Model 2: Adjusted for age and sex.

Model 3: Adjusted for age, sex, smoking status, alcohol consumption and household income.

Model 4: Adjusted for age, sex, smoking status, alcohol consumption, household income, body mass index, diabetes and prior fracture.

Model 5: Adjusted for age, sex, smoking status, alcohol consumption, household income, body mass index, diabetes, prior fracture and region.

### Association between changes in regular physical activity and hip fracture

We used Cox regression analysis to determine HRs for newly diagnosed hip fractures according to change in RPA status. The reference group was the always inactive group. The always active group (aHR: 0.691, 95% CI: 0.646–0.740) and the group that became active (aHR: 0.827; 95% CI: 0.787–0.870) exhibited significantly lower risks of hip fracture, despite adjustments for several potentially confounding variables (i.e., age, sex, smoking status, alcohol consumption, household income, BMI, diabetes, prior fracture and region). The always active group had the lowest incidence of hip fracture (Table 3).

**Table 3:** Hazard ratios for hip fracture according to change in regular physical activity in adults aged ≥ 40 years.

Group	Fracture event (n)	Total FU duration (PY)	IR (per 1,000 PY)	Hazard ratio (95% CI)				
				Model 1	Model 2	Model 3	Model 4	Model 5
Always inactive	12,237	14,014,911.3	0.87	1	1	1	1	1
Became inactive	1,873	2,483,882.1	0.75	0.863	0.843	0.859	0.865	0.861
				(0.822–0.906)	(0.803–0.886)	(0.818–0.902)	(0.824–0.908)	(0.820–0.905)
Became active	1,745	2,779,982.8	0.63	0.719	0.802	0.819	0.827	0.830
				(0.684–0.756)	(0.763–0.844)	(0.779–0.862)	(0.787–0.870)	(0.789–0.873)
Always active	928	1,914,666.5	0.48	0.555	0.651	0.679	0.691	0.691
				(0.520–0.594)	(0.609–0.696)	(0.635–0.726)	(0.646–0.740)	(0.646–0.740)

FU, follow-up; PY, person-year; IR, incidence rate; CI, confidence interval.

Incidence rate = Fracture event/total follow-up duration.

Model 1: Non-adjusted.

Model 2: Adjusted for age and sex.

Model 3: Adjusted for age, sex, smoking status, alcohol consumption and household income.

Model 4: Adjusted for age, sex, smoking status, alcohol consumption, household income, body mass index, diabetes and prior fracture.

Model 5: Adjusted for age, sex, smoking status, alcohol consumption, household income, body mass index, diabetes, prior fracture and region.

### Subgroup analysis

**Fig 2:**
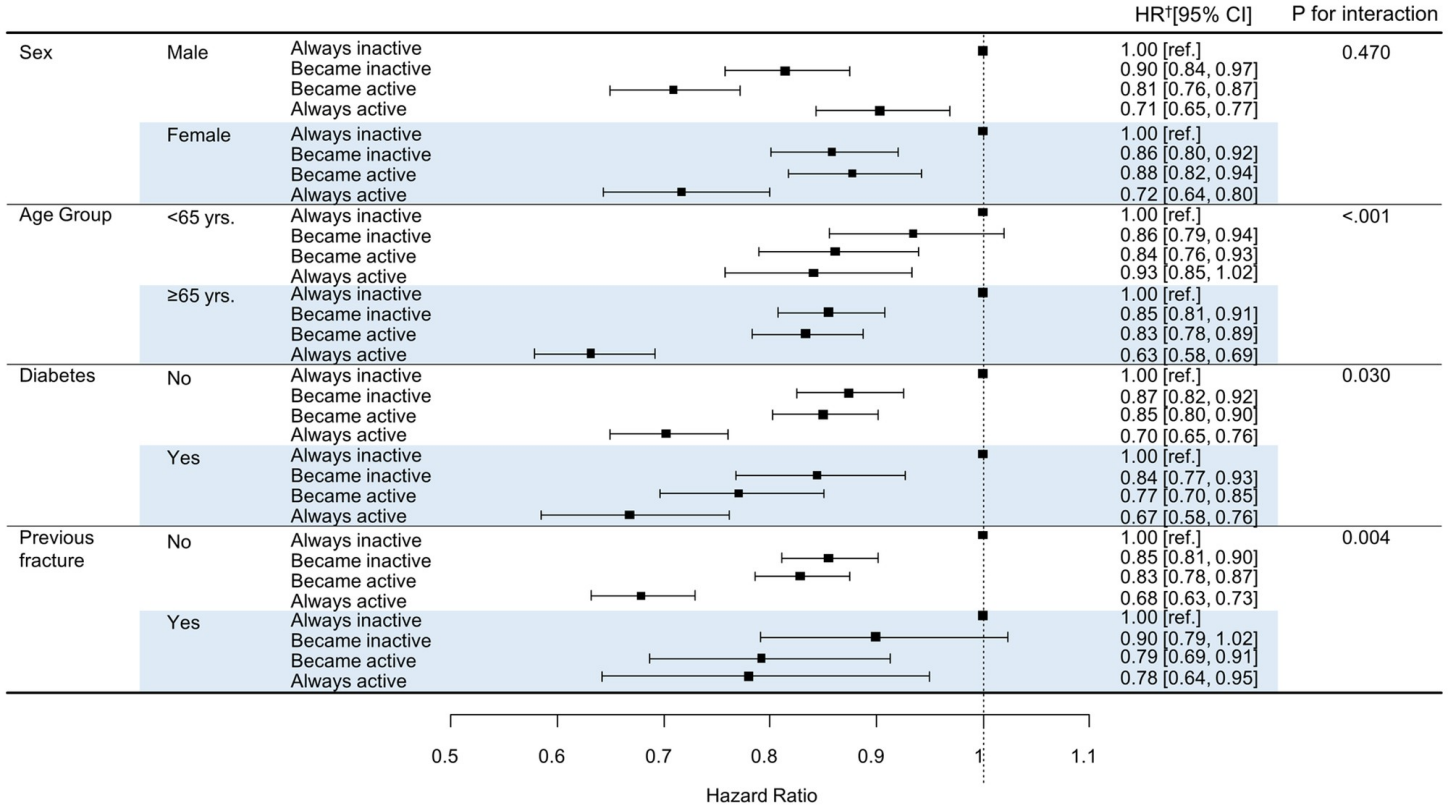
Associations between change in regular physical activity and risk of hip fracture by subgroup. †Model adjusted for age, sex, smoking status, alcohol consumption, income, body mass index, diabetes, and prior fracture. HR, hazard ratio; CI, confidence interval.

We performed subgroup analysis to explore possible effects of sex, age, BMI, smoking status, alcohol consumption, household income, hypertension, diabetes, dyslipidemia, CKD, and prior fracture on the risk of hip fracture. Always active participants aged ≥ 65 years who had diabetes and did not have prior fracture exhibited a significantly reduced risk of hip fracture, compared to always inactive participants (Fig 2). Younger age, BMI, smoking status, alcohol consumption, household income, hypertension, dyslipidemia, and CKD did not have any effects (all P>0.05).

## Discussion

We sought to characterize the relationship between changes in RPA status and hip fracture in the general population. Our principal finding was that always active participants (engaged in regular MVPA) exhibited a significantly lower risk of hip fracture than participants who were always inactive. This relationship was strongest among participants aged ≥ 65 years who had diabetes and no history of prior fracture.

### Regular physical activity and hip fracture

A physically active lifestyle affords personal benefits and reduces the risks of various chronic diseases from a public health perspective [10]. Many studies have shown that MVPA reduces the risks of various fractures, including hip fracture [8, 9, 14]. However, many individuals do not engage in adequate physical activity; only 31% of Americans do so [15]. We explored how RPA affected the risk of hip fracture. Simple leisure activities and low-level physical activity do not reduce this risk [16]. We found that consistent RPA was prophylactic. Multicomponent exercise programs that include MVPA have been shown to enhance the areal bone mineral density (BMD) of the femoral neck in middle-aged and older individuals [17]. Long-term exercise reduces the fall risk by enhancing the sense of balance [18]; because most hip fractures are caused by falls, a reduction in fall risk equates to a reduction in hip fracture risk [19]. Long-term, progressive resistance exercise improves muscle strength and morphology [20, 21]; muscle protects bone from severe trauma and stress [22].

### Hip fractures according to subgroup

Hip fracture prevention in the elderly is a public health challenge; mortality reaches 35% in patients aged ≥ 75 years and 45% of survivors exhibit functional decline [23]. We found that always active participants aged ≥ 65 years exhibited a much lower risk of hip fracture (HR: 0.63). Consistent RPA in older age is very beneficial but should be planned carefully. Resistance training increases the risk of musculoskeletal injury and may cause pain that interferes with daily life [24]. In individuals with diabetes, BMD can be reduced by oxidative stress; hyperglycemia; and the accumulation of advanced glycation end-products that enhance marrow adiposity, compromise collagen elasticity, and trigger the release of inflammatory factors and adipokines from visceral fat that may affect osteocyte function [25]. For patients with diabetes, the risk of fracture is reduced if the BMD is enhanced by means of regular exercise. We found that consistent RPA was more preventative of hip fracture in participants with diabetes than in participants without diabetes.

The preventative effect of consistent RPA was better in the group with no history of fracture than in the group with a history of fracture. However, regardless of prior fracture status, the preventative effect of RPA was better in the always active group than in the always inactive group; this suggests that individuals with prior fractures should be encouraged to engage in RPA. A meta-analysis revealed that a moderate amount of structured exercise after hip fracture improved overall mobility [26]. Thus, in individuals with a history of fracture, the amount of exercise should be carefully monitored. Clinicians may consider RPA to prevent hip fractures in people who are older, physically inactive, and exhibit diabetes accompanied by low BMD.

### Strengths and limitations

The principal strength of our study was that its sample size was very large and nationally representative. Additionally, this study involved analyses of data from two consecutive national health screens to explore the relationship between a change in RPA status and risk of hip fracture. To the best of our knowledge, this is the first study to identify an association between a change in RPA status and risk of hip fracture in a large Asian population. However, our study had certain limitations. First, the incidence of hip fractures may have been underestimated because NHIS data are only available for patients who sought medical services. In addition, bias may occur as the individuals without data on ICD-10 code and physical activity were excluded. Second, the intensity and frequency of physical activity were self-reported and may thus be inaccurate. However, the IPAQ-based self-reported physical activity questionnaire yielded valid data in prior studies [27]. Third, although Korea is rapidly becoming westernized, our findings should be generalized with caution; ethnicity and geography may have affected the results. Fourth, we did not consider patients with cognitive impairment; cognitive impairment and dementia are well-known risk factors for hip fracture [28]. Also, we did not consider the use of glucocorticoids and diagnosis of rheumatoid arthritis; these are potential confounders that are related with the hip fracture [29]. Therefore, further studies including these comorbid diseases and treatments are needed. Fifth, we did not consider osteoporosis-related hip fractures; we employed only the ICD-10 codes S72.0, S72.1, and S72.2. BMD affects the risk of fracture; notably, osteoporosis treatments reduce this risk [30]. Since there is no information on BMD in the NHIS database, we have not been able to analyze osteoporosis-related fractures. Sixth, we defined prior fracture as the case where the fracture occurred within 3 years from index year although the evidence suggests increased risk following a first fracture up to 10 years post-fracture [31]. This was because the NHIS database contains the data only from 2006. Seventh, we only analyzed the risk of fractures according to changes in RPA. However, some other factors can also be considered for the risk of fractures. Moderate levels of activity, including walking, can be associated with a lower risk of hip fracture and leashed dog walking was suggested to be related with increased fractures [32, 33]. Finally, it should be approached with caution when interpreting and applying our results clinically, and well-designed clinical trials are required to compensate for these limitations in the future.

## Conclusions

RPA was significantly associated with a reduced risk of hip fracture in the general population. Changes in RPA status were also related to the risk of hip fracture; consistent RPA imparted the maximum benefit.

## Supporting information

S1 Table Result of post-hoc analysis using Bonferroni correction about variables between groups. (DOCX)

S1 File ICD-10 code summary in this study. (DOCX)

S2 File STROBE checklist. (DOC)
