# Supplementary material for: Effects of changes in regular physical activity status on hip fracture: A nationwide population-based cohort study in Korea
Source: PLoS One. 2021 Apr 8;16(4):e0249819. doi: 10.1371/journal.pone.0249819 (PMC8031301; doi:10.1371/journal.pone.0249819)
Supplement: S1 Table — (DOCX) [file pone.0249819.s001.docx]

**S1 Table. Result of post-hoc analysis using Bonferroni correction about variables between groups.**

|  | **Pair** | **P-value** |
| --- | --- | --- |
| Male sex (%) | Always inactive – Became inactive | <.0001 |
|  | Always inactive – Became active | 1 |
|  | Always inactive – Always active | <.0001 |
|  | Became inactive – Became active | 1 |
|  | Became inactive – Always active | <.0001 |
|  | Became active – Always active | <.0001 |
| Age, years | Always inactive – Became inactive | <.0001 |
|  | Always inactive – Became active | <.0001 |
|  | Always inactive – Always active | <.0001 |
|  | Became inactive – Became active | <.0001 |
|  | Became inactive – Always active | <.0001 |
|  | Became active – Always active | <.0001 |
| Age ≥65 (%) | Always inactive – Became inactive | <.0001 |
|  | Always inactive – Became active | <.0001 |
|  | Always inactive – Always active | <.0001 |
|  | Became inactive – Became active | <.0001 |
|  | Became inactive – Always active | <.0001 |
|  | Became active – Always active | 1 |
| BMI, kg/m^2^ | Always inactive – Became inactive | <.0001 |
|  | Always inactive – Became active | <.0001 |
|  | Always inactive – Always active | <.0001 |
|  | Became inactive – Became active | <.0001 |
|  | Became inactive – Always active | 1 |
|  | Became active – Always active | <.0001 |
| BMI ≥25 (%) | Always inactive – Became inactive | <.0001 |
|  | Always inactive – Became active | <.0001 |
|  | Always inactive – Always active | <.0001 |
|  | Became inactive – Became active | <.0001 |
|  | Became inactive – Always active | <.0001 |
|  | Became active – Always active | <.0001 |
| Current smoker (%) | Always inactive – Became inactive | <.0001 |
|  | Always inactive – Became active | <.0001 |
|  | Always inactive – Always active | <.0001 |
|  | Became inactive – Became active | 0.0072 |
|  | Became inactive – Always active | <.0001 |
|  | Became active – Always active | <.0001 |
| Current drinker (%) | Always inactive – Became inactive | <.0001 |
|  | Always inactive – Became active | <.0001 |
|  | Always inactive – Always active | <.0001 |
|  | Became inactive – Became active | <.0001 |
|  | Became inactive – Always active | <.0001 |
|  | Became active – Always active | <.0001 |
| Low income (%) | Always inactive – Became inactive | 0.0012 |
|  | Always inactive – Became active | <.0001 |
|  | Always inactive – Always active | <.0001 |
|  | Became inactive – Became active | <.0001 |
|  | Became inactive – Always active | <.0001 |
|  | Became active – Always active | <.0001 |
| Hypertension (%) | Always inactive – Became inactive | <.0001 |
|  | Always inactive – Became active | <.0001 |
|  | Always inactive – Always active | <.0001 |
|  | Became inactive – Became active | <.0001 |
|  | Became inactive – Always active | <.0001 |
|  | Became active – Always active | <.0001 |
| Diabetes (%) | Always inactive – Became inactive | <.0001 |
|  | Always inactive – Became active | <.0001 |
|  | Always inactive – Always active | <.0001 |
|  | Became inactive – Became active | <.0001 |
|  | Became inactive – Always active | <.0001 |
|  | Became active – Always active | <.0001 |
| Dyslipidemia (%) | Always inactive – Became inactive | <.0001 |
|  | Always inactive – Became active | <.0001 |
|  | Always inactive – Always active | <.0001 |
|  | Became inactive – Became active | <.0001 |
|  | Became inactive – Always active | <.0001 |
|  | Became active – Always active | 1 |
| CKD (%) | Always inactive – Became inactive | <.0001 |
|  | Always inactive – Became active | 0.3795 |
|  | Always inactive – Always active | 0.0003 |
|  | Became inactive – Became active | <.0001 |
|  | Became inactive – Always active | <.0001 |
|  | Became active – Always active | 0.2667 |
| Urban region (%) | Always inactive – Became inactive | <.0001 |
|  | Always inactive – Became active | <.0001 |
|  | Always inactive – Always active | <.0001 |
|  | Became inactive – Became active | <.0001 |
|  | Became inactive – Always active | <.0001 |
|  | Became active – Always active | <.0001 |
| Prior fracture (%) | Always inactive – Became inactive | 0.2595 |
|  | Always inactive – Became active | <.0001 |
|  | Always inactive – Always active | <.0001 |
|  | Became inactive – Became active | <.0001 |
|  | Became inactive – Always active | <.0001 |
|  | Became active – Always active | <.0001 |
| Hip fracture (%) | Always inactive – Became inactive | <.0001 |
|  | Always inactive – Became active | <.0001 |
|  | Always inactive – Always active | <.0001 |
|  | Became inactive – Became active | <.0001 |
|  | Became inactive – Always active | <.0001 |
|  | Became active – Always active | <.0001 |

BMI, body mass index; CKD, chronic kidney disease
