# Supplementary material for: Effects of changes in regular physical activity status on hip fracture: A nationwide population-based cohort study in Korea
Source: PLoS One. 2021 Apr 8;16(4):e0249819. doi: 10.1371/journal.pone.0249819 (PMC8031301; doi:10.1371/journal.pone.0249819)
Supplement: S1 File — (DOCX) [file pone.0249819.s002.docx]

**ICD-10 Code summary in this study**

**Diabetes:** E11-14

**Dyslipidemia**: E78

**Hip fracture:** S72.0, S72.1, and S72.2

**Hypertension:** I10-13 and I15
